# Supplementary material for: Large exchange-driven intrinsic circular dichroism of a chiral 2D hybrid perovskite
Source: Nat Commun. 2024 Mar 22;15:2573. doi: 10.1038/s41467-024-46851-2 (PMC10959982; doi:10.1038/s41467-024-46851-2)
Supplement: Supplementary file 1 — Supplementary Information [file 41467_2024_46851_MOESM1_ESM.pdf]

Supplementary Materials

Large exchange-driven intrinsic circular dichroism of a chiral 2D hybrid perovskite

Shunran Li[1,2,#], Xian Xu[2,3,#], Conrad A. Kocoj[1,2], Chenyu Zhou[4], Yanyan Li[1,2], Du Chen[1,2], Joseph A. Bennett[2,5], Sunhao Liu[6], Lina Quan[6,7], Suchismita Sarker[8], Mingzhao Liu[4], Diana Y. Qiu[2,3,*], Peijun Guo[1,2,*]

[1]*Department of Chemical and Environmental Engineering, Yale University, New Haven, CT 06520, USA*

[2]*Energy Sciences Institute, Yale University, West Haven, CT 06516, USA*

[3]*Department of Mechanical Engineering and Materials Science, Yale University, New Haven, CT 06520, USA*

[4]*Center for Functional Nanomaterials, Brookhaven National Laboratory, Upton, NY 11973, USA*

[5]*Department of Chemistry, Yale University, New Haven, CT 06520, USA*

[6]*Department of Chemistry, Virginia Tech, Blacksburg, VA 24061, USA*

[7]*Department of Materials Science and Engineering, Virginia Tech, Blacksburg, VA 24061, USA*

[8]*Cornell High Energy Synchrotron Source, Cornell University, Ithaca, NY 14853, USA*

[#]These authors contributed equally.

Corresponding authors: diana.qiu@yale.edu, peijun.guo@yale.edu

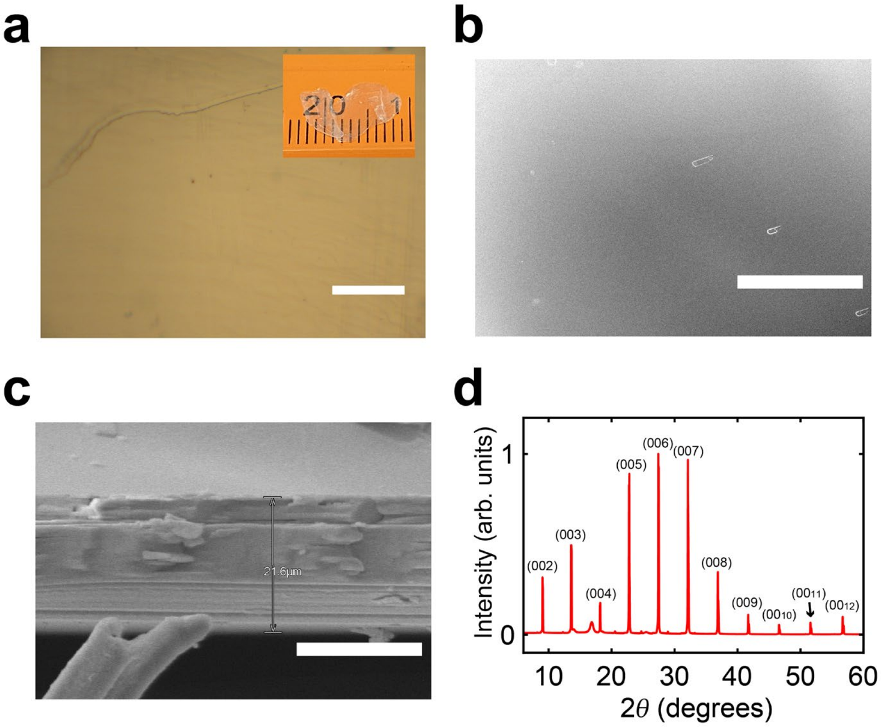

Supplementary Figure 1 | Structural and morphological characterization of *S*-NPB single crystals. a Optical micrograph of a representative *S*-NPB single crystal with centimeter dimensions (scale bar: 100 μm). **b-c** Scanning electron microscopy (SEM) images of an *S*-NPB crystal (top view in **b** and side view in **c**). The scale bar is 100 μm in **b** and 20 μm in **c**. **d** X-ray diffraction pattern of an *S*-NPB single crystal with characteristic out-of-plane peaks.

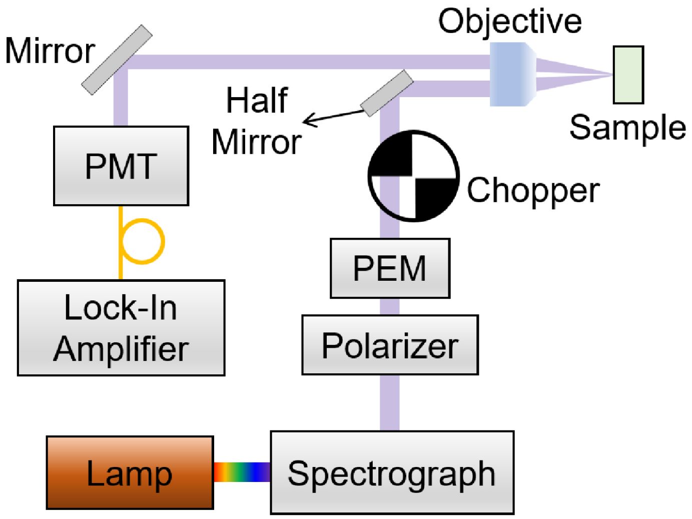

Supplementary Figure 2 | Experimental schematic. A schematic drawing of the customized optical microscopy setup used for the chiroptical micro-reflectance experiments. PMT: photomultiplier tube. PEM: photoelastic modulator.

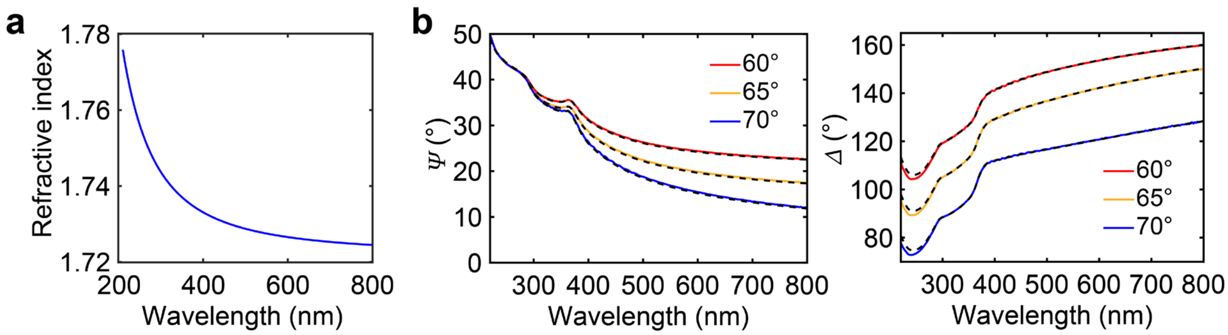

Supplementary Figure 3 | Refractive index characterization of the Al_2O_3 layer. a The real part of the RI of Al_2O_3 determined from spectroscopic ellipsometry. The imaginary part of RI of Al_2O_3 is identically zero. **b** Ψ and Δ spectra measured from spectroscopic ellipsometry measurements (color-solid lines) and their best fits (black-dashed lines).

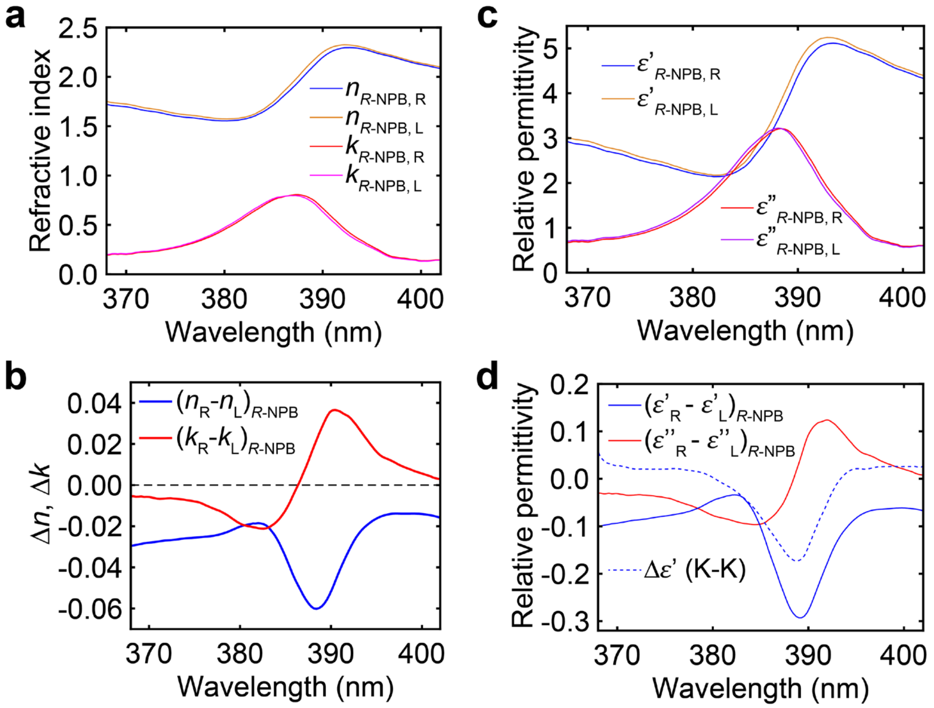

Supplementary Figure 4 | Circular polarization-resolved optical properties of *R*-NPB. a CP-dependent n and k for *R*-NPB. **b** Differences in n and k between RCP and LCP for *R*-NPB. **c** CP-dependent ε' and ε'' for *R*-NPB. **d** Differences in ε' and ε'' between RCP and LCP for *R*-NPB. The blue-dashed line is the change in ε' caused by the change in ε'' calculated using the Kramers-Kronig (K-K) relations.

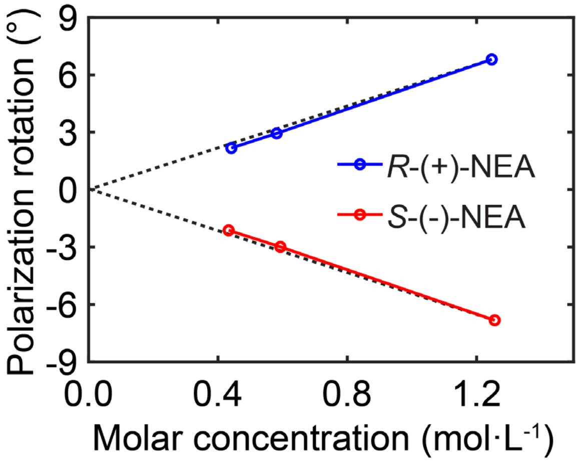

Supplementary Figure 5 | Degrees of optical polarization rotation induced by *S*-(-)-NEA and *R*-(+)-NEA molecules dissolved in ethanol at three different molar concentrations. The black-dashed lines are visual guides to the eyes demonstrating the linear dependence of the polarization rotation angle on the concentration of the chiral molecules. The measurements were performed with a Rudolph Autopol IV polarimeter. The wavelength of light used was 589 nm and the optical pathlength in the solution is 5 cm. By fitting the two lines, the optical rotation power is determined to be ±1.15 degrees·cm^{-1} (+ for *R*-NEA; − for *S*-NEA) at a concentration of 1 mol·L^{-1}.

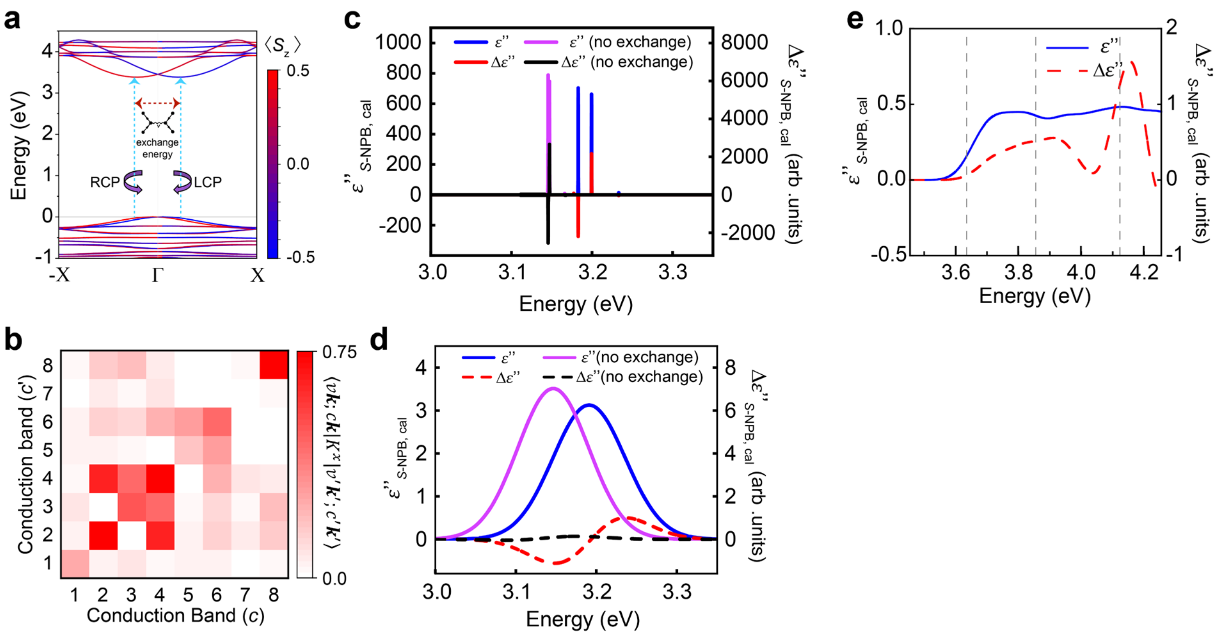

Supplementary Figure 6 | Correlation of electron-hole exchange interaction in the excitonic Cotton effect. a Spin-resolved DFT band structure of *S*-NPB with G_0W_0 bandgap obtained from a scissors correction and a schematic of the exchange coupling between two transitions on the RD-split states; the inset shows a Feynman diagram of the exchange term, and the transitions for the RCP and LCP light. RCP: right-hand circularly polarized light. LCP: left-hand circularly polarized light. **b** Electron-hole exchange matrix elements $\langle vc\boldsymbol{k}|K^x|v'c'\boldsymbol{k}'\rangle$ for $\boldsymbol{k} = 0.4X$, $\boldsymbol{k}' = 0.4X$ and $v = v' = v_1$ for *S*-NPB. **c** and **d** Imaginary parts of the (change in) relative permittivity, ε'' ($\Delta\varepsilon''$) for *S*-NPB with a small broadening coefficient of 0.1 meV in **c** and a sizeable broadening coefficient of 46 meV in **d**, respectively. The red and black curves represent $\Delta\varepsilon''$ with and without the exchange energy, respectively. **e** Differences in the relative permittivity without including excitonic effects for LCP and RCP light for *S*-NPB.

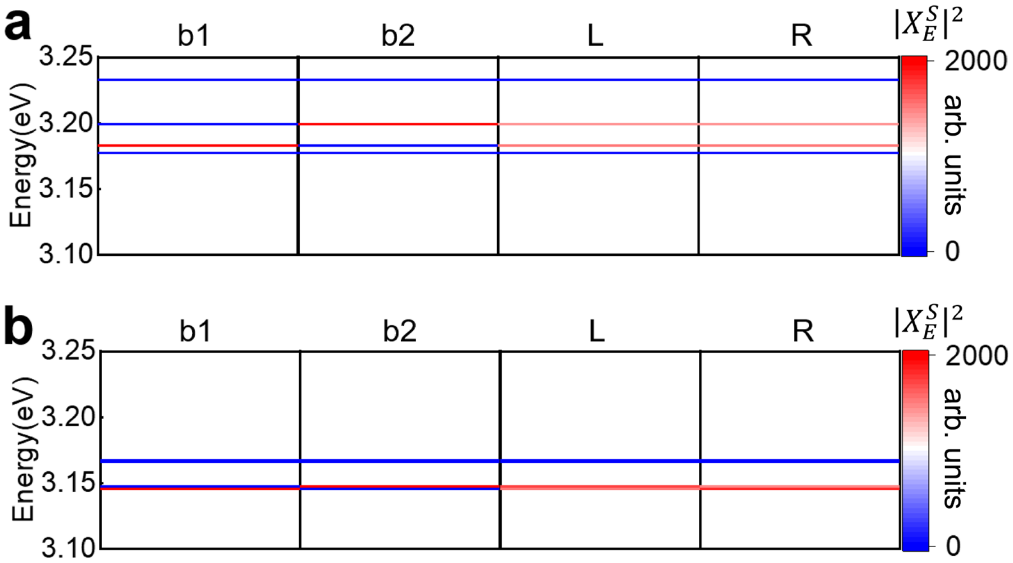

Supplementary Figure 7 | Spectrum of exciton eigen-energy levels for *S*-NPB. Spectrum of exciton eigen-energy levels for *S*-NPB, calculated with electron-hole exchange energy in **a** and without electron-hole exchange energy in **b**. In the figures, b1 and b2 stand for linearly polarized light with polarization in the $\Gamma - X$ (b1) and $\Gamma - Y$ (b2) directions, while L and R stand for LCP and RCP. The lines are colored according to the oscillator strength of each exciton state with the given polarizations of light. Bright states are red, and dark states are blue. Note that the bright in-plane excitons are split in panel **a**, but their splitting disappears in panel **b** once the exchange interaction is removed.

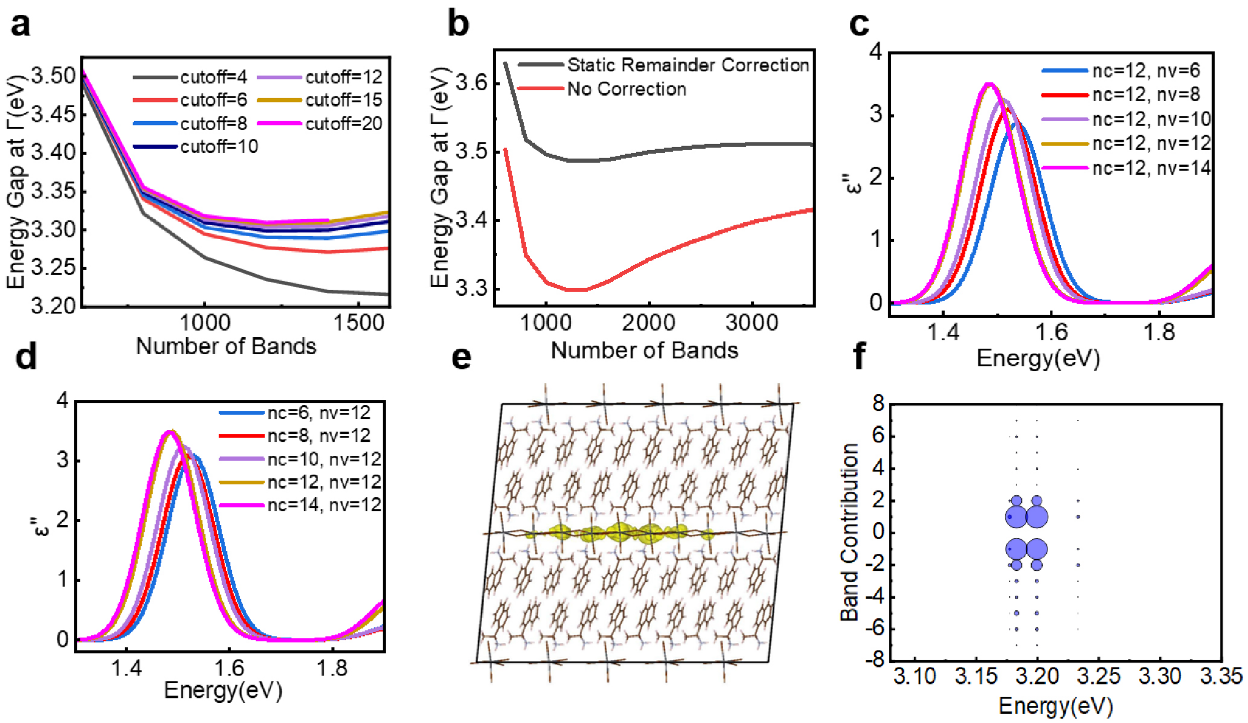

Supplementary Figure 8 | First-principles details. a Convergence of the G_0W_0 energy gap at the Γ point with respect to the total number of bands included in the sum over empty states in the calculation of the GW self-energy and the screened energy cutoff for the dielectric matrix. **b** Convergence of the G_0W_0 energy gap at the Γ point with respect to the total number of bands with and without the static remainder correction.[1] **c** and **d** Convergence of the imaginary part of the macroscopic dielectric function with the number of valance bands (n_v) in **c** and conduction bands (n_c) in **d**. **e** First bright exciton in the real space with a 1.42-nm radius and 0.46-eV binding energy. **f** Contribution of each band to each exciton state. The size of each dot corresponds to $f \times \sum_{n\boldsymbol{k}} |A^S_{vc\boldsymbol{k}}|$, where $A^S_{vc\boldsymbol{k}}$ is the electron-hole amplitude of the exciton state $|S\rangle = \sum_{vc\boldsymbol{k}} A^S_{vc\boldsymbol{k}} |vc\boldsymbol{k}\rangle$, $\boldsymbol{k}$ is a k-point, and $v(c)$ is the index of the hole (electron) state, as counted from the Fermi energy, contributing to the exciton state. For the conduction (valence) state contributions, n in the sum runs over valence (conduction) states. f is the oscillator strength of each exciton state under excitation by RCP light.

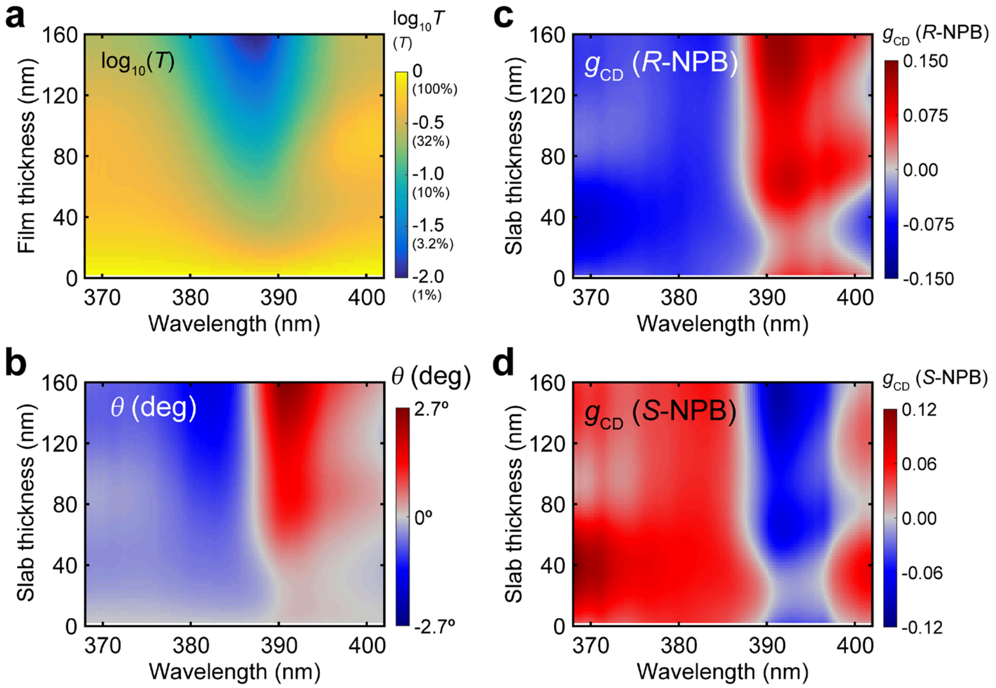

Supplementary Figure 9 | CD and dissymmetry data for R-NPB single crystals. a Calculated optical transmittance (averaged between LCP and RCP) for an *R*-NPB single-crystalline slab as a function of slab thickness and wavelength. The data is plotted in the $\log_{10}$ scale (numbers in parentheses show the transmittance values in a linear scale). **b** Calculated map of ellipticity θ (in degrees) as a function of wavelength and thickness of the single-crystalline *R*-NPB slab. **c** Anisotropy factor g_{CD} for an *R*-NPB single-crystalline slab as a function of slab thickness and wavelength. **d** Anisotropy factor g_{CD} for an *S*-NPB single-crystalline slab as a function of slab thickness and wavelength.

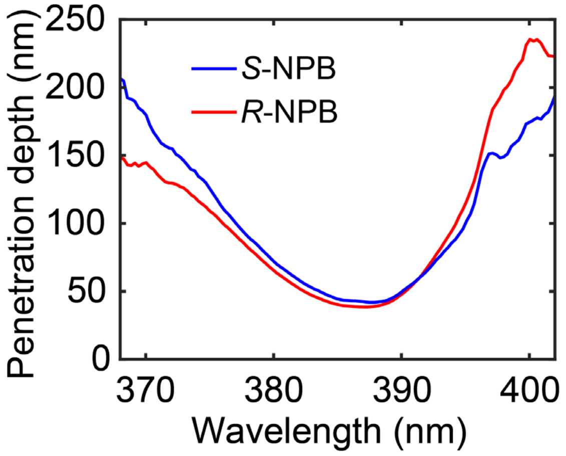

Supplementary Figure 10 | Calculated optical penetration depths for single crystals of *S*-NPB and *R*-NPB. The penetration depth is calculated as $1/\alpha$, where α is the absorption coefficient and is determined as $\alpha = \frac{2\pi\varepsilon''}{n\lambda}$. The differences between the two curves likely arise from quality variations between samples as well as experimental errors.

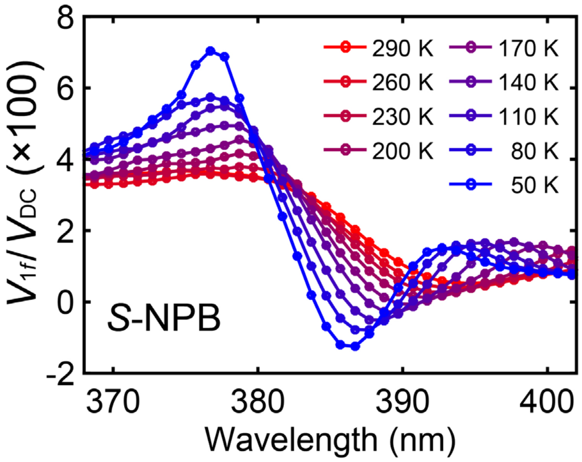

Supplementary Figure 11 | Temperature-dependent chiroptical reflectance experiments. The ratios of $1f$ voltage (V_{1f}) and DC voltage (V_{DC}) signals for an *S*-NPB single crystal measured in reflection at varying temperatures from 50 K to 290 K.

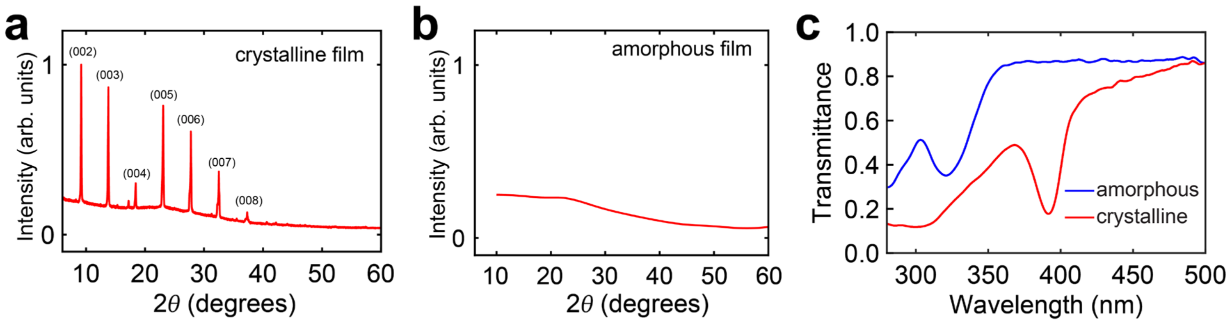

Supplementary Figure 12 | Structural characterization for *S*-NPB thin film samples. X-ray diffraction patterns of a spin-coated crystalline *S*-NPB film after annealing (in **a**) and before annealing (in **b**). **c** Transmittance spectra of spin-coated *S*-NPB films on sapphire substrates both before annealing (amorphous) and after annealing (crystalline).

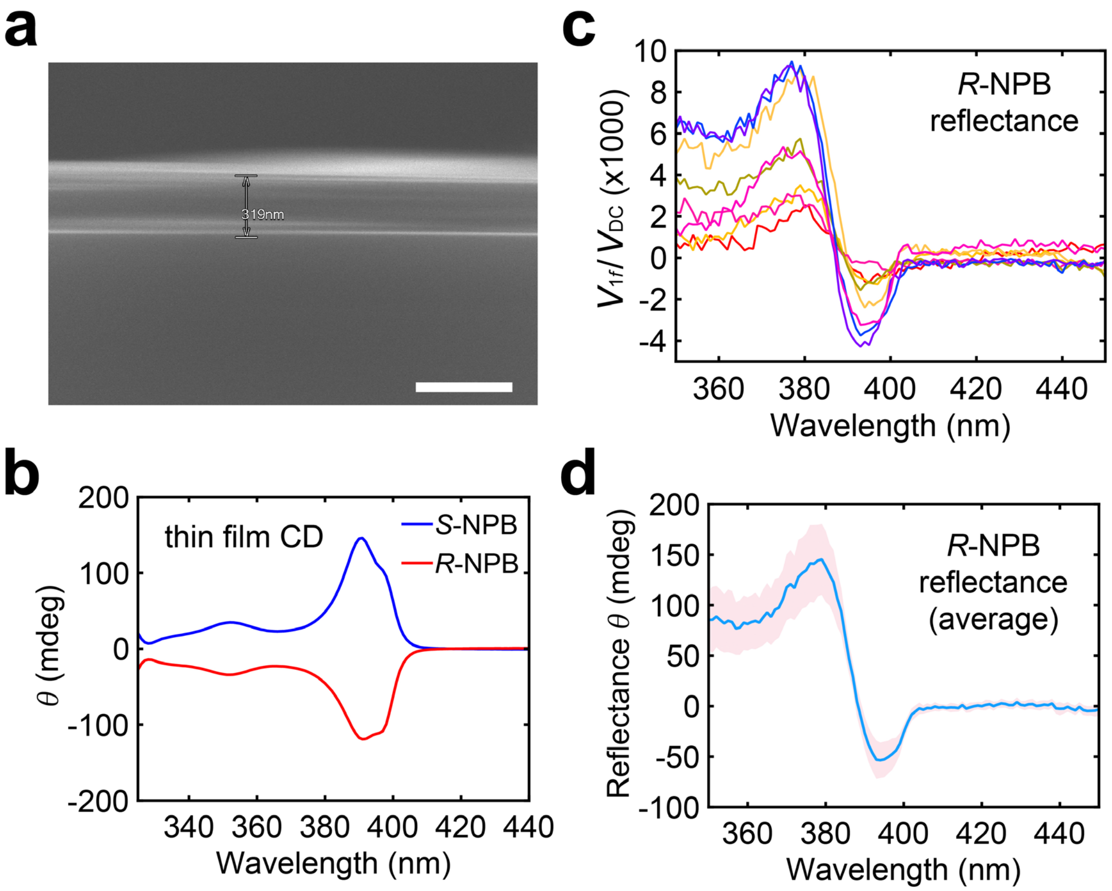

Supplementary Figure 13 | CD results on an annealed, crystalline *S*/*R*-NPB film. **a** Cross-sectional SEM image showing the thickness (~320 nm) of an *S*-NPB film. The scale bar is 500 nm. **b** CD spectra of *S*-NPB and *R*-NPB films measured using a commercial Applied Photophysics Chirascan CD spectrometer. **c** Ratios of the $1f$ voltage (V_{1f}) and DC voltage (V_{DC}) signals as a function of wavelength measured with the lock-in amplifier for an *R*-NPB film. **d** Plots of the average and standard deviation of ellipticity angle θ in reflectance, calculated using the data shown in **c**. The shaded area in **d** represents the standard deviation of the data shown in **c**.

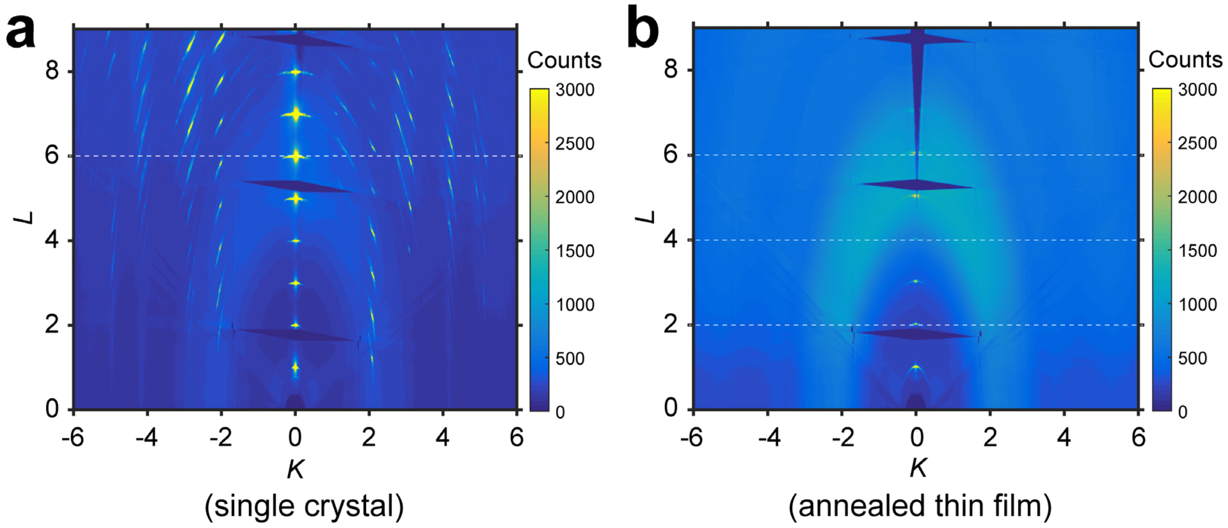

Supplementary Figure 14 | Synchrotron X-ray diffraction data for *S*-NPB. Synchrotron X-ray diffraction patterns at the $H = 0$ plane measured for an *S*-NPB single crystal in **a** and an annealed crystalline *S*-NPB thin film on glass substrate in **b**, showing the drastic difference in the crystallinity of the two types of samples.

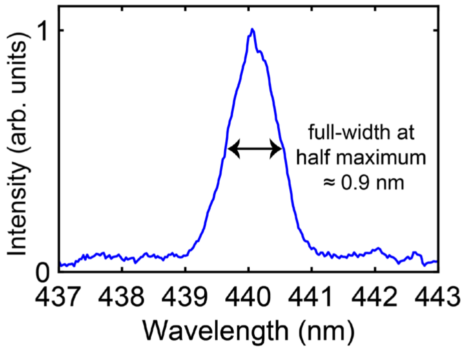

Supplementary Figure 15 | Spectral resolution of the CD reflectance setup. Representative spectrum of the monochromatic light output from the spectrograph showing a spectral resolution (in terms of full width at half maximum) higher than 1 nm. The spectrum was measured using a separate spectrograph (Kymera 328i, Andor) and an EMCCD camera (iXon Life 888, Andor) with a spectral resolution of approximately 0.1 nm.

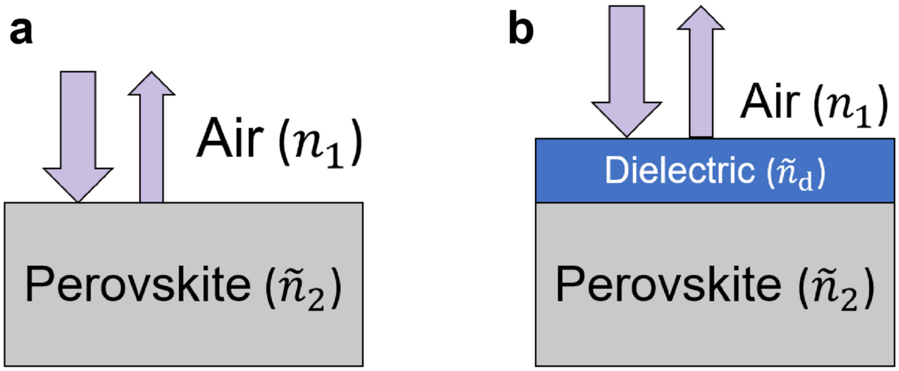

Supplementary Figure 16 | Schematic drawings of the reflectance calculation. Schematic of the incident light from medium 1 (air) reflected off a perovskite crystal in **a**, and reflected off a dielectric/perovskite crystal stack in **b**. The optically absorbing perovskite crystal with tens of µm in thickness can be considered as infinitely thick.

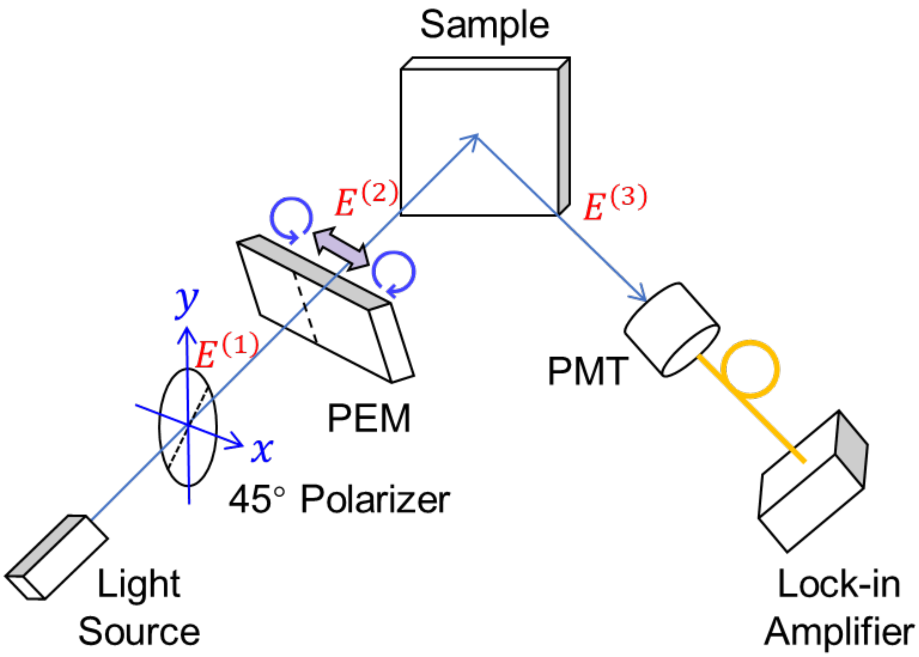

Supplementary Figure 17 | Schematic diagram. The CP-resolved reflection setup showing the essential optical components and the coordinate system used in the derivation. PMT: photomultiplier tube. PEM: photoelastic modulator.

Supplementary Note 1. Determination of the complex refractive index under unpolarized light

Step I. For an air/perovskite stack (Supplementary Fig. 16a), the complex reflection coefficient for normal incidence of a linearly polarized light can be calculated as

$$r_{12} = \frac{n_1 - \tilde{n}_2}{n_1 + \tilde{n}_2} \tag{1}$$

, where $n_1 = 1$ is the RI of air, and $\tilde{n}_2$ is the complex RI of the perovskite. The experimentally measured reflectivity is equal to $|r_{12}|^2$.

Step II. When the perovskite crystal is covered by a dielectric layer (*i.e.*, Al_2O_3 in this work), an air/dielectric/perovskite stack is formed (Supplementary Fig. 16b). The complex reflection coefficient for the stack under normal-incidence condition is

$$r = \frac{r_{1\mathrm{d}} + r_{\mathrm{d}2} e^{2i\beta}}{1 + r_{1\mathrm{d}} r_{\mathrm{d}2} e^{2i\beta}} \tag{2}$$

, where $r_{1\mathrm{d}} = \frac{n_1 - \tilde{n}_\mathrm{d}}{n_1 + \tilde{n}_\mathrm{d}}$, $r_{\mathrm{d}2} = \frac{\tilde{n}_\mathrm{d} - \tilde{n}_2}{\tilde{n}_\mathrm{d} + \tilde{n}_2}$, and $\beta = \frac{2\pi}{\lambda} \tilde{n}_\mathrm{d} h$. Note that $\tilde{n}_\mathrm{d}$ and h are the complex RI and thickness of the dielectric coating, respectively, which are determined from spectroscopic ellipsometry measurements on a silicon 'witness' wafer. Supplementary Fig. 3 shows the best fits to the RI of the 17-nm thick Al_2O_3 layer, as well as the associated ellipsometry data and their best fits.

Supplementary Note 2. Determination of the circular polarization-dependent refractive index

Reflectivity of chiral 2D-MHPs without a dielectric coating

We use the Jones matrix formulism and assume the incident light has an electric-field amplitude denoted by E. As shown in Supplementary Fig. 17, after the linear polarizer, the electric field becomes $E^{(1)} = \frac{E}{\sqrt{2}}\begin{bmatrix}1\\1\end{bmatrix}$, where $\hat{x} = \begin{bmatrix}1\\0\end{bmatrix}$ and $\hat{y} = \begin{bmatrix}0\\1\end{bmatrix}$ correspond to the unit vectors pointing along x and y, respectively. After the light passes the PEM, the electric field becomes $E^{(2)} = \frac{E}{\sqrt{2}}\begin{bmatrix}1\\e^{-i\delta}\end{bmatrix}$, where $\delta = \delta_0\sin(2\pi ft)$ is the retardation induced by the PEM. Here δ_0 is the amplitude of the retardation (set to $\frac{\pi}{2}$) and f is the modulation frequency (~50 kHz).

Transforming from the Cartesian to the circular coordinate, we can define circularly polarized light as

$$\hat{\mathrm{R}} = \frac{1}{\sqrt{2}}(\hat{x} - i\hat{y}) = \frac{1}{\sqrt{2}}\begin{bmatrix}1\\-i\end{bmatrix}, \text{ and } \hat{\mathrm{L}} = \frac{1}{\sqrt{2}}(\hat{x} + i\hat{y}) = \frac{1}{\sqrt{2}}\begin{bmatrix}1\\i\end{bmatrix}.$$

Then, we can rewrite $E^{(2)}$ as

$$E^{(2)} = \frac{E}{\sqrt{2}}\begin{bmatrix}1\\e^{-i\delta}\end{bmatrix} = \frac{E}{2}\left[\left(1 + ie^{-i\delta}\right)\hat{\mathrm{R}} + \left(1 - ie^{-i\delta}\right)\hat{\mathrm{L}}\right] \quad (3).$$

After reflection by the chiral perovskite, the electric field becomes

$$E^{(3)} = \frac{E}{2}\left[\left(1 + ie^{-i\delta}\right)\tilde{r}_{\mathrm{R}}\hat{\mathrm{R}} + \left(1 - ie^{-i\delta}\right)\tilde{r}_{\mathrm{L}}\hat{\mathrm{L}}\right] \quad (4),$$

where $\tilde{r}_{\mathrm{R}} = r_{\mathrm{R}}e^{i\theta_{\mathrm{R}}}$ and $\tilde{r}_{\mathrm{L}} = r_{\mathrm{L}}e^{i\theta_{\mathrm{L}}}$ are the complex reflection coefficients.

Transforming equation (4) back to the Cartesian coordinate yields

$$E^{(3)} = \frac{E}{2\sqrt{2}}\begin{bmatrix}(\tilde{r}_{\mathrm{R}} + \tilde{r}_{L}) + ie^{-i\delta}(\tilde{r}_{\mathrm{R}} - \tilde{r}_{\mathrm{L}})\\ -i(\tilde{r}_{\mathrm{R}} - \tilde{r}_{\mathrm{L}}) + e^{-i\delta}(\tilde{r}_{\mathrm{R}} + \tilde{r}_{\mathrm{L}})\end{bmatrix} \quad (5)$$

The square of the electric-field amplitude can then be calculated as

$$\left|E^{(3)}\right|^2 = \frac{E^2}{2}\left[(|r_{\mathrm{R}}|^2 + |r_{\mathrm{L}}|^2) + \sin\delta(|r_{\mathrm{R}}|^2 - |r_{\mathrm{L}}|^2)\right] \quad (6)$$

If the PEM is not modulating, we have $\delta = 0$, and $\left|E^{(3)}\right|^2 = \frac{E^2}{2}(|r_\mathrm{R}|^2 + |r_\mathrm{L}|^2)$.

With the PEM modulation, the square of the electric field amplitude becomes

$$\left|E^{(3)}\right|^2 = \frac{E^2}{2}\{(|r_\mathrm{R}|^2 + |r_\mathrm{L}|^2) + \sin[\delta_0 \sin(2\pi f t)](|r_\mathrm{R}|^2 - |r_\mathrm{L}|^2)\}$$

$$= \frac{E^2}{2}\{(R_\mathrm{R} + R_\mathrm{L}) + \sin[\delta_0 \sin(2\pi f t)](R_\mathrm{R} - R_\mathrm{L})\} \quad (7)$$

The derivation of $\left|E^{(3)}\right|^2$ above follows the approach reported in an earlier literature on magneto-optic Kerr effect (MOKE).[2] Note that $|r_\mathrm{R}|^2$ and $|r_\mathrm{L}|^2$ are denoted by R_R and R_L, which are the reflectivity of the material to RCL and LCP light, respectively.

To obtain both $R_\mathrm{R} + R_\mathrm{L}$ and $R_\mathrm{R} - R_\mathrm{L}$, we can perform a Jacobi-Anger expansion on the sine term,

$$\sin[\delta_0 \sin(2\pi f t)] = 2\sum_{n=1}^{\infty} J_{2n-1}(\delta_0) \cdot \sin[(2n-1)(2\pi f t)],$$

where $J_n(x)$ is the *n*-th order Bessel function.

When $\delta_0 = \frac{\pi}{2}$, $J_1(\delta_0) : J_3(\delta_0) : J_5(\delta_0) : J_7(\delta_0) = 0.5667 : 0.0689 : 0.0022 : 3.37 \times 10^{-5}$. Ignoring $J_7(\delta_0)$ and higher order terms, which have diminishing contribution, we can rewrite $\left|E^{(3)}\right|^2$ as

$$\left|E^{(3)}\right|^2 = \frac{E^2}{2}\{(R_\mathrm{R} + R_\mathrm{L}) + [1.1334 \cdot \sin(2\pi f t) + 0.1378 \cdot \sin(6\pi f t) + 0.0044 \cdot \sin(10\pi f t)](R_\mathrm{R} - R_\mathrm{L})\}$$

Based on the above expression for $\left|E^{(3)}\right|^2$, in the lock-in experiments, up to a common numerical constant $\frac{E^2}{2}$, the DC component $I_\mathrm{DC} \propto (R_\mathrm{R} + R_\mathrm{L})$, and the $1f$ component $I_{1f} \propto 1.1334(|R_\mathrm{R} - R_\mathrm{L}|)$, from which we can obtain the relationship,

$$\frac{R_\mathrm{R}}{R_\mathrm{L}} = \frac{2}{1 - \frac{1}{1.1334}\frac{I_{1f}}{I_\mathrm{DC}}} - 1 = \frac{2}{1 - 0.882\frac{I_{1f}}{I_\mathrm{DC}}} - 1 \text{ [if } R_\mathrm{R} - R_\mathrm{L} > 0 \text{ and hence } I_{1f} \propto 1.1334(R_\mathrm{R} - R_\mathrm{L})]$$

or

$$\frac{R_\mathrm{R}}{R_\mathrm{L}} = \frac{2}{1 + \frac{1}{1.1334}\frac{I_{1f}}{I_\mathrm{DC}}} - 1 = \frac{2}{1 + 0.882\frac{I_{1f}}{I_\mathrm{DC}}} - 1 \text{ [if } R_\mathrm{R} - R_\mathrm{L} < 0 \text{ and hence } I_{1f} \propto 1.1334(R_\mathrm{L} - R_\mathrm{R})]$$

As a result, by measuring $\frac{I_{1f}}{I_{\mathrm{DC}}}$ from the lock-in experiments, we can obtain the ratio of R_{R} and R_{L}. Note that a monochromator was used in conjunction with the PEM (Supplementary Fig. 2), and the lock-in detection was performed wavelength-by-wavelength. The relative amplitude of R_{R} and R_{L} was determined by comparing the phases of the $1f$ signal detected by the lock-in amplifier between **1)** the *S*-NPB or *R*-NPB sample, and **2)** a silver mirror followed by a $\frac{1}{4}\lambda$ waveplate and a linear polarizer set to passing RCP light but rejecting LCP light. We found that the *R*-NPB sample exhibited the same phase in the lock-in as the combination of (silver mirror/$\frac{1}{4}\lambda$ waveplate/linear polarizer), hence the light reflected by *R*-NPB has a stronger RCP component and a weaker LCP component. Due to the inversion of circular polarization upon reflection, the *R*-NPB sample has a stronger (weaker) R_{L} (R_{R}), therefore its $\frac{R_{\mathrm{R}}}{R_{\mathrm{L}}} = \frac{2}{1-0.882\frac{I_{1f}}{I_{\mathrm{DC}}}} - 1$. In contrast, the *S*-NPB sample has a stronger (weaker) R_{R} (R_{L}), and therefore its $\frac{R_{\mathrm{R}}}{R_{\mathrm{L}}} = \frac{2}{1+0.882\frac{I_{1f}}{I_{\mathrm{DC}}}} - 1$. Corroborated by the absolute value of $(R_{\mathrm{L}} + R_{\mathrm{R}})/2$ determined from reflectance measurements with un-polarized light (Fig. 1c), the R_{L} and R_{R} for *S*/*R*-NPB (before the dielectric coating) can be determined individually.

As shown in equation (1), $r_{\mathrm{R/L}} = \frac{n_1 - \tilde{n}_{\mathrm{R/L}}}{n_1 + \tilde{n}_{\mathrm{R/L}}}$, with $\tilde{n}_{\mathrm{R}}$ and $\tilde{n}_{\mathrm{L}}$ the complex RI of the perovskite for RCP and LCP, respectively; *i.e.*, $\tilde{n}_{\mathrm{R}} = n_{\mathrm{R}} + ik_{\mathrm{R}}$ and $\tilde{n}_{\mathrm{L}} = n_{\mathrm{L}} + ik_{\mathrm{L}}$. It is noted that the $R_{\mathrm{R}} = |r_{\mathrm{R}}|^2$ only depends on $\tilde{n}_{\mathrm{R}}$, and $R_{\mathrm{L}} = |r_{\mathrm{L}}|^2$ only depends on $\tilde{n}_{\mathrm{L}}$.

<u>Reflectivity of chiral 2D-MHPs with a dielectric coating and determination of $\tilde{n}_R$ and $\tilde{n}_L$</u>

The only difference between this case and the case discussed above (*i.e.*, the case without the dielectric coating) is that the reflection coefficient $r_{\mathrm{R/L}}$ needs to be calculated using equation (2), *i.e.*, R_{R} (R_{L}) is calculated from equation (2) by letting $n_3 = \tilde{n}_{\mathrm{R}}$ ($n_3 = \tilde{n}_{\mathrm{L}}$). By performing reflectivity measurements on the chiral 2D-MHPs both before and after the coating, we can obtain $R_{R,\mathrm{before}}$ and $R_{R,\mathrm{after}}$ at each wavelength, from which we can obtain $\tilde{n}_R = n_R + ik_R$ (two input parameters for two unknowns at each wavelength), and likewise obtain $\tilde{n}_L = n_L + ik_L$.

Supplementary Note 3. Circular polarization-dependent permittivity in solid-state systems

CP-dependent permittivity in the independent-particle picture

To the lowest order, the coupling to the electromagnetic field is described as

$$H_{\text{int}} = -\frac{e}{m_e}\hat{\boldsymbol{p}} \cdot \boldsymbol{A} \tag{8}$$

Here, $\hat{\boldsymbol{p}}$ and $\boldsymbol{A}$ are, respectively, the momentum operator and vector potential. The vector potential is

$$\boldsymbol{A} = |\boldsymbol{A}_0| \sum_{\boldsymbol{k}} \hat{\epsilon} \left(e^{i\boldsymbol{k}\cdot\boldsymbol{r}}\hat{a}_{\boldsymbol{k}} + e^{-i\boldsymbol{k}\cdot\boldsymbol{r}}\hat{a}_{\boldsymbol{k}}^{\dagger}\right) \tag{9}$$

where $\hat{\epsilon}$ is the polarization direction, and $\hat{a}_{\boldsymbol{k}}$ ($\hat{a}_{\boldsymbol{k}}^{\dagger}$) is the annihilation (creation) operator of the vector potential. For small fields, one can expand $e^{i\boldsymbol{k}\cdot\boldsymbol{r}} = (1 + i\boldsymbol{k}\cdot\boldsymbol{r} + \cdots)$. Keeping terms to second order in the expansion, the interaction Hamiltonian is[3]

$$H_{\text{int}} = -\frac{e}{m_e}\hat{\boldsymbol{p}} \cdot \boldsymbol{A}_0 - \frac{e}{2m_e}\hat{\boldsymbol{L}} \cdot (\nabla \times \boldsymbol{A}_0) + H_{\text{eq}} \tag{10}$$

, where the first term corresponds to the electric dipole, the second term corresponds to the magnetic dipole due to the angular momentum operator $\hat{\boldsymbol{L}} = \hat{\boldsymbol{r}} \times \hat{\boldsymbol{p}}$ (neglecting the spin angular momentum contribution), H_{eq} corresponds to the electric quadrupole term, and $\boldsymbol{A}_0$ is the vector potential at the unperturbed nuclear coordinate. Typically, when looking at linear absorption in the low-field limit, the magnetic dipole and electric quadrupole terms are much smaller than the electric dipole term and can thus be neglected. However, in the context of CP-dependent permittivity, the magnetic term becomes crucial for breaking the time-reversal symmetry, and the CD effect (or equivalently the differential absorption of LCP and RCP light) depends on the cross-terms of the electric and magnetic dipoles. Applying Fermi's Golden Rule and neglecting the electric quadrupole term, we can obtain the imaginary part of the dielectric function in the independent-particle basis:[4]

$$\varepsilon''(\omega) = \frac{8\pi^2 e^2}{\omega^2}\sum_{vc\boldsymbol{k}} \left|X_E^{vc\boldsymbol{k}} + X_M^{vc\boldsymbol{k}}\right|^2 \delta(\omega - \omega_{vc\boldsymbol{k}}) \quad (11)$$

, where $X_E^{vc\boldsymbol{k}} = \hat{A} \cdot \langle v\boldsymbol{k}|\hat{\boldsymbol{p}}|c\boldsymbol{k}\rangle$ and $X_M^{vc\boldsymbol{k}} = \frac{1}{2}\langle v\boldsymbol{k}|\hat{\boldsymbol{L}} \cdot (\nabla \times \hat{A})|c\boldsymbol{k}\rangle$ are the electric and magnetic dipole matrix elements, respectively, v (c) is the index of the valence (conduction) band, $\boldsymbol{k}$ is a k-point in the 1st Brillouin zone, $\hat{A} = \boldsymbol{A}/|\boldsymbol{A}|$ is the direction of the vector potential, and $\omega_{vc\boldsymbol{k}}$ is the energy of the independent-particle transition between the valance and conduction bands at point $\boldsymbol{k}$. For thin film materials at normal incidence, the polarization directions of LCP and RCP can be written as $\hat{\epsilon}_{\mathrm{L}} = (\hat{x} + i\hat{y})/\sqrt{2}$ and $\hat{\epsilon}_{\mathrm{R}} = (\hat{x} - i\hat{y})/\sqrt{2}$.

For the simplicity of the formula, we decompose electric and magnetic dipole matrix elements according to the particular directions of the vector potential:

$$(X_E^x)_{vc\boldsymbol{k}} = \langle v\boldsymbol{k}|\hat{p}_x|c\boldsymbol{k}\rangle A_x/|\boldsymbol{A}|$$

$$(X_E^y)_{vc\boldsymbol{k}} = \langle v\boldsymbol{k}|\hat{p}_y|c\boldsymbol{k}\rangle A_y/|\boldsymbol{A}|$$

$$(X_M^x)_{vc\boldsymbol{k}} = \frac{1}{2}\langle v\boldsymbol{k}|\hat{L}_y|c\boldsymbol{k}\rangle \frac{\partial A_x}{\partial z}/|\boldsymbol{A}|$$

$$(X_M^y)_{vc\boldsymbol{k}} = -\frac{1}{2}\langle v\boldsymbol{k}|\hat{L}_x|c\boldsymbol{k}\rangle \frac{\partial A_y}{\partial z}/|\boldsymbol{A}|$$

As a result, the difference in $\varepsilon''(\omega)$ between LCP and RCP, $\varepsilon''_{\mathrm{L}}(\omega) - \varepsilon''_{\mathrm{R}}(\omega)$, is

$$\begin{aligned}\Delta\varepsilon''(\omega) &= \frac{8\pi^2 e^2}{\omega^2}\sum_{vc\boldsymbol{k}}\left(X_E^{L^*}X_M^L + X_E^L X_M^{L^*} - X_E^{R^*}X_M^R - X_E^R X_M^{R^*}\right)_{vc\boldsymbol{k}}\delta(\omega - \omega_{vc\boldsymbol{k}}) \\ &= \frac{8\pi^2 e^2 i}{\omega^2}\sum_{vc\boldsymbol{k}}\left(-X_E^x X_M^{y^*} + X_E^y X_M^{x^*} - X_M^x X_E^{y^*} + X_M^y X_E^{x^*}\right)_{vc\boldsymbol{k}}\delta(\omega - \omega_{vc\boldsymbol{k}}) \\ &= \frac{16\pi^2 e^2}{\omega^2}\sum_{vc\boldsymbol{k}}\mathrm{Im}\left(X_E^x X_M^{y^*} - X_E^y X_M^{x^*}\right)_{vc\boldsymbol{k}}\delta(\omega - \omega_{vc\boldsymbol{k}}) \quad (12)\end{aligned}$$

These findings above align with the prior studies on CD of chiral molecules, where the CD was found to be proportional to the electric dipole-magnetic dipole cross terms.[5,6] However, unlike that in molecular systems, the position operator $\hat{r}$ is challenging to calculate in the Bloch basis of periodic solid-state systems, requiring a numerical derivative of Kohn-Sham wavefunctions with

an arbitrary phase. Therefore, to calculate the angular momentum, we assume that nonlocal contribution is negligible in the unperturbed Hamiltonian H_0, resulting in $[H_0, \hat{\boldsymbol{r}}] = \left[-\frac{\hat{p}^2}{2m} + V(\vec{r}), \hat{\boldsymbol{r}}\right] = \frac{i\hbar\hat{\boldsymbol{p}}}{m}$. This enables the conversion of all position matrix elements:

$$\langle \boldsymbol{k}m|\hat{\boldsymbol{r}}|\boldsymbol{k}n\rangle = \frac{i\hbar}{m_e}\frac{\langle \boldsymbol{k}m|\hat{\boldsymbol{p}}|\boldsymbol{k}n\rangle}{E_{m\boldsymbol{k}} - E_{n\boldsymbol{k}}} \tag{13}$$

We can then apply a sum-over-states method to calculate the angular momentum matrix elements from the momentum matrix elements:[7,8,9]

$$L_x(\boldsymbol{k}; m, n) = \frac{i\hbar}{m_e}\sum_{n'}\left(\frac{\langle \boldsymbol{k}m|p_y|\boldsymbol{k}n'\rangle\langle \boldsymbol{k}n'|p_z|\boldsymbol{k}n\rangle}{E_{m\boldsymbol{k}} - E_{n'\boldsymbol{k}}} - \frac{\langle \boldsymbol{k}m|p_z|\boldsymbol{k}n'\rangle\langle \boldsymbol{k}n'|p_y|\boldsymbol{k}n\rangle}{E_{m\boldsymbol{k}} - E_{n'\boldsymbol{k}}}\right) \tag{14}$$

L_y and L_z can be calculated in a similar fashion.

CP-dependent permittivity including exciton effects

The independent-particle picture is insufficient to describe the 2D MHPs with strongly bound excitons, and therefore it is necessary to include electron-hole interactions to account for excitonic effects. The exciton eigenstates were calculated through the solution of the BSE in the Tamm-Dancoff approximation (TDA):[4]

$$(E_{c\boldsymbol{k}} - E_{v\boldsymbol{k}})A^S_{vc\boldsymbol{k}} + \sum_{v'c'\boldsymbol{k}'}\langle vc\boldsymbol{k}|K^{eh}|v'c'\boldsymbol{k}'\rangle A^S_{v'c'\boldsymbol{k}'} = \Omega^S A^S_{vc\boldsymbol{k}} \tag{15}$$

Here, $A^S_{vc\boldsymbol{k}}$, K^{eh}, and Ω^S are the electron-hole amplitude corresponding to the excited state $|S\rangle = \sum_{vc\boldsymbol{k}} A^S_{vc\boldsymbol{k}}|vc\boldsymbol{k}\rangle$, the electron-hole interaction kernel, and the exciton excitation energy, respectively. Then, the electric-dipole transition and magnetic-dipole transition between the excited state S and the ground state 0 can be defined:

$$\langle 0|\hat{\boldsymbol{p}}|S\rangle = \sum_{vc\boldsymbol{k}} A^S_{vc\boldsymbol{k}}\langle v\boldsymbol{k}|\hat{\boldsymbol{p}}|c\boldsymbol{k}\rangle$$

$$\langle 0|\hat{\boldsymbol{L}}|S\rangle = \sum_{vc\boldsymbol{k}} A^S_{vc\boldsymbol{k}}\langle v\boldsymbol{k}|\hat{\boldsymbol{L}}|c\boldsymbol{k}\rangle$$

With a method similar to the independent-particle case, we define $X_E^S = \langle 0|\hat{\boldsymbol{p}}|S\rangle \cdot \hat{\epsilon}$ and $X_M^S = \frac{1}{2}\langle 0|\hat{\boldsymbol{L}}|S\rangle \cdot (\nabla \times \hat{A})$. Applying Fermi's Golden Rule, we obtain

$$\varepsilon''(\omega) = \frac{8\pi^2 e^2}{\omega^2} \sum_S |X_E^S + X_M^S|^2 \delta(\omega - \Omega^S) \tag{16}$$

Hence, the difference in $\varepsilon''(\omega)$ between LCP and RCP is

$$\begin{aligned}
\Delta\varepsilon''(\omega) &= \frac{8\pi^2 e^2}{\omega^2} \sum_S \left(X_E^{S,L^*} X_M^{S,L} + X_E^{S,L} X_M^{S,L^*} - X_E^{S,R^*} X_M^{S,R} - X_E^{S,R} X_M^{S,R^*} \right) \delta(\omega - \Omega^S) \\
&= \frac{8\pi^2 e^2 i}{\omega^2} \sum_S \left(-X_E^{S,x} X_M^{S,y^*} + X_E^{S,y} X_M^{S,x^*} - X_M^{S,x} X_E^{S,y^*} + X_M^{S,y} X_E^{S,x^*} \right) \delta(\omega - \Omega^S) \\
&= \frac{16\pi^2 e^2}{\omega^2} \sum_S \mathrm{Im} \left(X_E^{S,x} X_M^{S,y^*} - X_E^{S,y} X_M^{S,x^*} \right) \delta(\omega - \Omega^S)
\end{aligned} \tag{17}$$

The corresponding $\Delta\varepsilon'(\omega)$ can be calculated using the Kramers-Kronig relations.

The excitonic peak in the absorption spectrum corresponds to two bright exciton states with similar oscillator strength separated by an energy of 17 meV. These exciton states originate from the transitions between the lowest conduction band and the highest valence band, as marked in Supplementary Fig. 6a. As discussed in the main text, the highest valence bands and the lowest conduction bands are RD-split states, which originate from the inorganic lead-bromide framework. Such splitting results from asymmetric octahedral distortions induced by the chiral organic spacers and the pronounced spin-orbit coupling exhibited by lead. In an independent-particle picture, these two transitions should have the same energy and are dipole-allowed for LCP and RCP, respectively. However, the independent particle picture cannot capture the 17-meV energy splitting and the accompanying Cotton effect.

We included the exciton effects by solving the BSE. In the BSE, the electron-hole interaction kernel K^{eh} consists of a direct interaction K^d and an exchange interaction K^x:[4]

$$\langle v\boldsymbol{k}; c\boldsymbol{k}|K^d|v'\boldsymbol{k}'; c'\boldsymbol{k}'\rangle = -\sum_{\boldsymbol{G}\boldsymbol{G}'} M_{cc'}^*(\boldsymbol{k},\boldsymbol{q},\boldsymbol{G}) W_{\boldsymbol{G}\boldsymbol{G}'}(\boldsymbol{q}) M_{vv'}(\boldsymbol{k},\boldsymbol{q},\boldsymbol{G}') \tag{18}$$

$$\langle v\boldsymbol{k}; c\boldsymbol{k}|K^x|v'\boldsymbol{k}'; c'\boldsymbol{k}'\rangle = \sum_{\boldsymbol{G}} M_{cv}(\boldsymbol{k}, 0, \boldsymbol{G})v(\boldsymbol{G})M^*_{cv'}(\boldsymbol{k}', 0, \boldsymbol{G}) \tag{19}$$

Here, $W_{\boldsymbol{GG}'}(\boldsymbol{q})$ and $v(\boldsymbol{G})$ are the screened and bare Coulomb interactions, respectively; $\boldsymbol{G}$ is the reciprocal lattice vector; $\boldsymbol{q} = \boldsymbol{k} - \boldsymbol{k}'$; and M is the matrix element with $M^*_{nn'}(\boldsymbol{k}, \boldsymbol{q}, \boldsymbol{G}) = \langle n\boldsymbol{k}|e^{i(\boldsymbol{q}+\boldsymbol{G})\cdot\boldsymbol{r}}|n'\boldsymbol{k}'\rangle$. We see that the direct interaction is large when the electron-electron or the hole-hole overlap is significant, while the exchange interaction scales with the electron-hole overlap. Consequently, the exchange can couple electron-hole pairs of different character including electron-hole pairs on different RD-split bands. The exchange matrix elements $\langle v\boldsymbol{k}; c\boldsymbol{k}|K^x|v'\boldsymbol{k}'; c'\boldsymbol{k}'\rangle$ coupling two k-points, where $\boldsymbol{k}' = -\boldsymbol{k}$ are shown in Supplementary Fig. 6b. It is seen that the coupling of the two RD-split states, corresponding to the matrix element $c = c' = 1$, is nonzero. Such nonzero exchange coupling is responsible for the 17-meV splitting of the band-edge bright exciton states. When the exchange interaction is turned off, the splitting of the states is reduced to approximately 1 meV (Supplementary Fig. 6c).

The exchange-split states have opposite selection rules for LCP and RCP light. As a result, when the states are broadened, the associated $\Delta\varepsilon''$ exhibits a derivative-like line-shape, which is reminiscent of the Cotton effect. In the absence of exchange energy and with a realistic broadening, the contributions to CD from the nearly degenerate states with a 1-meV separation cancel each other out, resulting in negligible CD (Supplementary Fig. 6d).

Finally, we show that the derivative-like line-shape at the band edge is purely an excitonic effect that disappears in the noninteracting limit. We calculated the $\Delta\varepsilon''$ spectrum without electron-hole interactions (Supplementary Fig. 6e). The three vertical dashed lines indicate 1) the direct bandgap (arising from the inorganic octahedra), 2) the smallest bandgap between the third valence band (which derives from the organic spacers) and the lowest conduction band, and 3) the smallest gap associated with transitions from the highest valence band to the third conduction band (from the organic spacers). We see that there is still a small amount of CD coming from the band-to-band transitions between the highest valence and the lowest conduction states between the first two dashed lines. Although $\Delta\varepsilon''$ is nonzero in the plotted spectral range, the Cotton effect completely disappears, which shows that the observed Cotton effect in experiment relies on both many-body exciton effects and the exchange energy among exciton states. We list several necessary conditions

for the formation of the exchange-driven Cotton effect: a breaking of the inversion symmetry, strong spin-orbit coupling, the first few conduction bands and valence bands are isolated RD-split bands, a strongly bound and isolated 1st exciton peak, a finite exchange energy among the relevant exciton states, and an overall chiral space group.

Supplementary References

1. Deslippe J, Samsonidze G, Jain M, Cohen ML, Louie SG. Coulomb-hole summations and energies for *GW* calculations with limited number of empty orbitals: A modified static remainder approach. *Phys. Rev. B* **87**, 165124 (2013).
2. Sato K. Measurement of magneto-optical Kerr effect using piezo-birefringent modulator. *Jpn. J. Appl. Phys.* **20**, 2403 (1981).
3. Wang J, Dong H, Li S-W. Magnetic dipole-dipole interaction induced by the electromagnetic field. *Phys. Rev. A* **97**, 013819 (2018).
4. Deslippe J, Samsonidze G, Strubbe DA, Jain M, Cohen ML, Louie SG. BerkeleyGW: A massively parallel computer package for the calculation of the quasiparticle and optical properties of materials and nanostructures. *Comput. Phys. Commun.* **183**, 1269-1289 (2012).
5. Warnke I, Furche F. Circular dichroism: electronic. *WIREs Comput. Mol. Sci.* **2**, 150-166 (2012).
6. Govorov AO, Fan Z, Hernandez P, Slocik JM, Naik RR. Theory of circular dichroism of nanomaterials comprising chiral molecules and nanocrystals: plasmon enhancement, dipole interactions, and dielectric effects. *Nano Lett.* **10**, 1374-1382 (2010).
7. Deilmann T, Krüger P, Rohlfing M. Ab Initio studies of exciton *g* factors: monolayer transition metal dichalcogenides in magnetic fields. *Phys. Rev. Lett.* **124**, 226402 (2020).
8. Amit T, Hernangómez-Pérez D, Cohen G, Qiu DY, Refaely-Abramson S. Tunable magneto-optical properties in MoS_2 via defect-induced exciton transitions. *Phys. Rev. B* **106**, L161407 (2022).
9. Xuan F, Quek SY. Valley Zeeman effect and Landau levels in two-dimensional transition metal dichalcogenides. *Phys. Rev. Res.* **2**, 033256 (2020).
